# Research progress on the chemical components and biological activities of sea cucumber polypeptides

**DOI:** 10.3389/fphar.2023.1290175

**Published:** 2023-10-16

**Authors:** Yiwen Shou, Chao Feng, Qinpei Lu, Xin Mao, Huisha Huang, Zhiheng Su, Hongwei Guo, Zhaoquan Huang

**Affiliations:** ^1^ Guangxi Key Laboratory for Bioactive Molecules Research and Evaluation and College of Pharmacy, Guangxi Medical University, Nanning, Guangxi, China; ^2^ Key Laboratory of Longevity and Aging-related Diseases of Chinese Ministry of Education and Center for Translational Medicine, Guangxi Medical University, Nanning, Guangxi, China; ^3^ Department of Pathology, The First Affiliated Hospital of Guangxi Medical University, Nanning, Guangxi, China

**Keywords:** sea cucumber, polypeptides, chemical composition, biological activity, anti-cancer

## Abstract

Owing to their unique physical and chemical properties and remarkable biological activities, marine biological resources are emerging as important sources of raw materials for producing health products, food, and cosmetics. Collagen accounts for approximately 70% of the sea cucumber body wall, and its hydrolysis produces small-molecule collagen polypeptides with diverse biological functions, such as anticancer, antihypertensive, immune-enhancing, memory-enhancing, and cartilage tissue repairing effects. Notably, the potential of sea cucumber polypeptides in combination with anticancer therapy has garnered considerable attention. Determining the composition and structure of sea cucumber polypeptides and exploring their structure–activity relationships will aid in obtaining an in-depth understanding of their diverse biological activities and provide scientific insights for the development and utilization of these polypeptides. Therefore, this review focuses on the amino acid structures and activities of sea cucumber polypeptides of varying molecular weights. This study also provides an overview of the biological activities of various sea cucumber polypeptides and aims to establish a scientific basis for their development.

## 1 Introduction

Sea cucumbers are echinoderms inhabiting intertidal zones and shallow marine environments. There are over 1,700 species of sea cucumbers worldwide (Pangestuti and Arifin, 2018). In China, approximately 140 species have been identified, most of which are distributed in the temperate and tropical regions. Temperate sea cucumbers are primarily found in the Yellow and Bohai Seas, whereas tropical sea cucumbers are concentrated in the coastal areas of the Guangdong, Guangxi, and Hainan provinces in China (Li and Chang, 2006). Comparative studies on sea cucumber composition have revealed that sea cucumbers from various species and sources exhibit shared characteristics, such as their high protein and low fat contents. Their amino acid compositions are also quite similar (Li and Chang, 2006).

**FIGURE 1:**
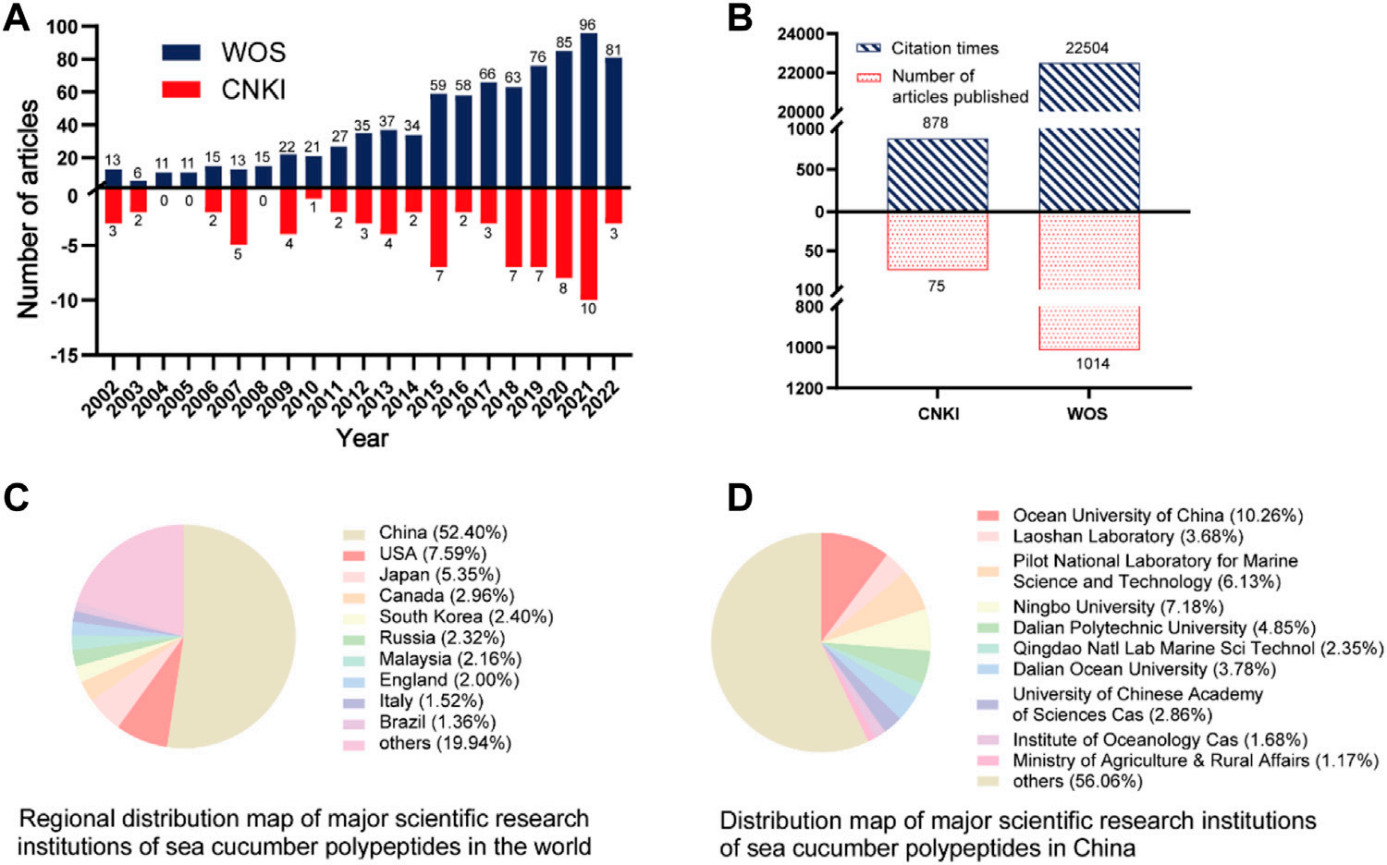
Related studies on sea cucumber polypeptides published in the last 20 years: **(A)** The number of publications on sea cucumber polypeptides, according to statistics from the Web of Science (WOS) and China National Knowledge Infrastructure (CNKI), over the past two decades; **(B)** The total number of studies and citations related to sea cucumber polypeptides in the CNKI and WOS in the last 20 years; **(C)** From 2002 to 2022, the regional distribution map of major scientific research institutions investigating sea cucumber polypeptides in the world was collected on WOS; **(D)** From 2002 to 2022, the distribution map of major scientific research institutions investigating sea cucumber polypeptides in China was recorded on WOS.

**FIGURE 2:**
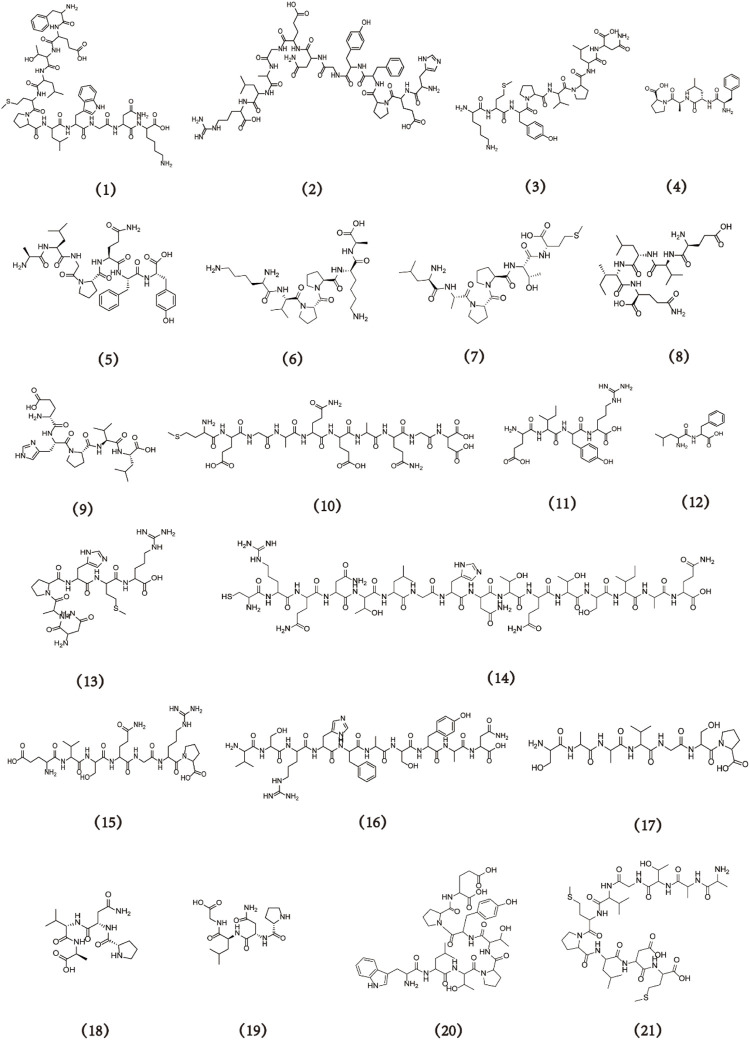
The structural formula of sea cucumber peptides (**1–21**).

**FIGURE 3:**
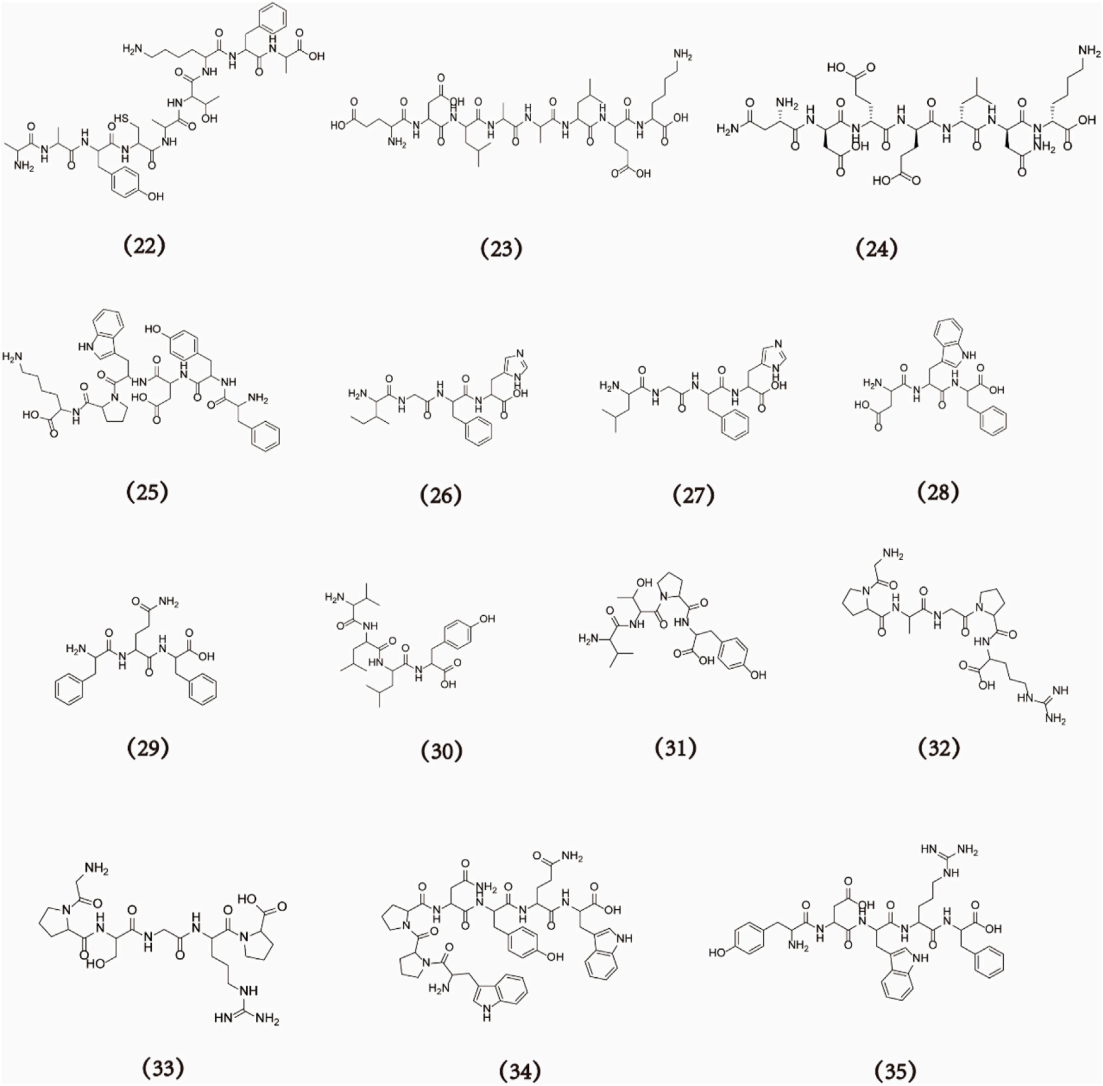
The structural formula of sea cucumber peptides (**22–35**).

Sea cucumbers possess biologically active components, such as sea cucumber collagen, polypeptides, polysaccharides, and saponins (Yang et al., 2022). Sea cucumber polypeptides, specifically small-molecule peptides with molecular weights below 2 kDa, have attracted widespread attention because of their potential applications in the development of functional medicine and drugs (Thomas et al., 2016; Thomas et al., 2017). According to statistics from the Web of Science and China National Knowledge Infrastructure, the number of publications on sea cucumber polypeptides has consistently increased over the past two decades. By the end of 2022, more than 1,000 papers about sea cucumber polypeptides were published, and the cumulative number of citations exceeded 20,000. Furthermore, literature collected from the WOS database suggests that Chinese research institutions, especially Ocean University of China, Ningbo University, and the Pilot National Laboratory for Marine Science and Technology, have made considerable contributions to sea cucumber polypeptide research (Figure 1). This article summarises the biological activities of sea cucumber polypeptides with different molecular weights. In addition, the sources, preparation processes, purity, molecular formula, and structure of these polypeptides (Figures 2, 3) as well as the potential molecular mechanisms of action of different bioactive sea cucumber peptides (Supplementary Table S1) are discussed to provide a resource for future studies on sea cucumber polypeptides.

## 2 Biological activities of sea cucumber polypeptides

Sea cucumber polypeptides may be obtained by the enzymatic hydrolysis of sea cucumber collagen. These polypeptides are known for their safety, high biological activities, and strong specificity. Sea cucumber-derived active peptides have been reported to possess diverse functional properties, including antioxidative, blood pressure-lowering, metal ion-chelating, neuroprotective, wound healing, uric acid-lowering, antitumor, anti-fatigue, hypoglycaemic, bone-promoting, collagen-boosting, anti-inflammatory, and immunomodulatory effects. Most studies have focused on investigating the extraction and preparation methods and biological effects of sea cucumber polypeptides and active peptide sequences. This research has had remarkable implications in the advancement of sea cucumber-based functional foods and the modulation of human physiological processes by such products.

### 2.1 Antioxidative activity

Oxidative stress occurs when the antioxidant defence system of the body cannot counterbalance the production of oxidants, thereby resulting in pathological alterations, including neurodegeneration, wound ulceration, and cancer. The development of novel peptides with antioxidative properties, particularly a new generation of natural antioxidants, represents a considerable advancement in improving the ability of the body to defend itself against oxidative stress.

In a sequential hydrolysis process, trypsin and papain were used to prepare *Apostichopus japonicus* protein hydrolysate (AjPH) with a high peptide content (Guo et al., 2020). Three peptides were identified from AjPH: FETLMPLWGNK (**1**) (C_63_H_94_N_14_O_16_S, 1,336 Da), HEPFYGNEGALR (**2**) (C_62_H_88_N_18_O_19_, 1,389 Da), and KMYPVPLN (**3**) (C_45_H_72_N_10_O_11_S, 961 Da). Under oxidative stress, all three peptides exhibit antioxidative activity by significantly restoring depleted glutathione levels, decreasing mitochondrial superoxide levels, and reducing mitochondrial autophagy (Lu et al., 2021a). HEPFYGNEGALR was found to activate the Nrf2/HO-1 pathway and inhibit nuclear translocation, thereby alleviating acute alcohol-induced liver injury resulting from oxidative stress and inflammatory reactions (Zhu et al., 2022).

Sea cucumbers are critical for peptide production. The body walls of *Cucumaria frondosa*, *Stichopus japonicus*, and *Parastichopus californicus* can effectively eliminate hydroxyl (OH·) and 2,2-diphenyl-1-picrylhydrazyl (DPPH) radicals and superoxide anions when enzymatically hydrolysed under identical conditions, suggesting that the antioxidative capacity of sea cucumber peptides varies across different species (Zhao et al., 2012). The type of enzyme used to hydrolyse the peptides is also crucial. For example, after using alcalase and trypsin to hydrolyse *C. frondosa*, a study utilising ultra-performance liquid chromatography (UPLC) found that peptide fragments with A<2 and T < 2 kDa had elevated Leu residue levels. Additionally, the essential amino acid/non-essential amino acid (EAA/NEAA) ratio of the A<2 kDa peptide fragment was higher than that of the T < 2 kDa peptide fragment. The A<2 and T < 2 kDa peptide fragments showed higher antioxidative activity *in vitro* than the other peptide segments by effectively eliminating DPPH and superoxide anion radicals (Zhang et al., 2020). In addition to commonly used enzyme sources, endogenous enzymes obtained from sea cucumbers are frequently used for protein hydrolysis. Self-digesting enzymes extracted from sea cucumber tentacles were used to hydrolyse the body walls of *Isostichopus fuscus*. Antioxidant peptide components with a molecular weight <3 kDa and strong capacity to scavenge oxygen-free radicals have been isolated using ultrafiltration and semi-preparative high-performance liquid chromatography (HPLC) techniques (Hernández-Sámano and Hernández-Ledesma, 2015). The components derived from the self-digestion of *Stichopus japonicus* have been found to exhibit a scavenging capacity against three reactive oxygen species (ROS) and displayed a dose-dependent relationship as the molecular weight increased (Wang et al., 2010). “Sea cucumber peptides can also modulate the activity of superoxide dismutase (SOD) and glutathione peroxidase (GSH-Px), and reduce oxidative stress in mouse serum and organs (Zhou et al., 2018).

Microwave radiation can be considered a viable alternative to traditional enzymatic methods for enhancing the efficiency of enzymatic hydrolysis. A microwave-assisted neutral enzyme technique was used to hydrolyse collagen from *Acaudina molpadioides* and successfully isolated the antioxidant peptide FLAP (**4**) using ultrafiltration, gel filtration chromatography, and reversed-phase high performance liquid chromatography (RP-HPLC) purification. FLAP has a molecular formula of C_23_H_34_N_4_O_5_, a molecular weight of 446 Da, and exhibits high DPPH radical scavenging activity (Jin et al., 2019). The incorporation of the non-protein amino acid cysteine into FLAP has been found to significantly increase the OH scavenging activity of the peptide, resulting in the strong inhibition of H_2_O_2_-induced oxidative damage, activation of the JNK/Nrf2 pathway, and mitigation of oxidative damage (Jin et al., 2021). Under identical conditions, the enzyme–alkali method has been used to enzymatically hydrolyse *A. molpadioides* and produce collagen peptides (CPs). These CPs have demonstrated the ability to scavenge OH and DPPH radicals in a dose-dependent manner. Furthermore, they reduce H_2_O_2_-induced ROS damage in mouse leukaemia cells and monocyte RAW264.7 macrophages (Li et al., 2019). Therefore, CPs could enhance the antioxidant defence capabilities of the human body.

### 2.2 Blood pressure lowering activity

Angiotensin-converting enzyme (ACE) is a target of hypertension treatment. Promoting the conversion of angiotensin I to angiotensin II in the renin-angiotensin or endocrine system is expected to reduce blood pressure by inhibiting excessive activity (Wilson et al., 2011).

Sea cucumber peptides exhibit potent antihypertensive properties. Bromelain was used as an enzymatic agent to hydrolyse *Actinopyga lecanora* and obtained five peptides using ultrafiltration and semi-preparative RP-HPLC techniques. The peptides included ALGPQFY (**5**) (C_39_H_54_N_8_O_10_, 794 Da), KVPPKA (**6**) (C_30_H_54_N_8_O_7_, 639 Da), LAPPTM (**7**) (C_29_H_52_N_6_O_8_S, 629 Da), EVLIQ (**8**) (C_27_H_48_N_6_O_9_, 601 Da), and EHPVL (**9**) (C_27_H_43_N_7_O_8_, 594 Da), all of which exhibit antihypertensive properties. These peptides are also associated with ACE inhibition (Auwal et al., 2019). The enzymatic hydrolysis of *A. lecanora* using alkaline proteases has been found to yield protein hydrolysates with dual antioxidative and antihypertensive functions (Ghanbari et al., 2015). The efficacy of these hydrolysates is directly proportional to their concentration. The alkaline protease hydrolysis of *Acaudina molpadioidea* yields PNVA (**18**) (C_17_H_29_N_5_O_6_, 399 Da) and PNLG (**19**) (C_17_H_29_N_5_O_6_, 399 Da), which exhibit antihypertensive effects related to ACE inhibition (Li et al., 2018b).

Enzyme combinations are commonly used to produce sea cucumber peptides. In a previous study, the body wall proteins of *A. molpadioides* were subjected to continuous hydrolysis using pineapple and alkaline proteinases to obtain peptides with high ACE inhibitory activity. Protein hydrolysate II (PH-II, <2 kDa) was purified and isolated using various techniques, such as ultrafiltration, ion-exchange chromatography, size-exclusion chromatography, and RP-HPLC. This process resulted in the isolation of an ACE-inhibiting peptide, MEGAQEAQGD (**10**), with a molecular formula of C_39_H_62_N_12_O_19_S and molecular weight of 1,034 Da. At a concentration of 3 mmol.L^–1^.kg^–1^, treatment with this peptide significantly lowered blood pressure in spontaneously hypertensive rats (Zhao et al., 2009). Alternatively, composite proteinases have been used to enzymatically hydrolyse *S. horrens*, resulting in the extraction and separation of four peptides using ultrafiltration, RP-HPLC, electrophoresis, and electrospray ionization quadrupole time-of-flight tandem mass spectrometry (ESI-Q-TOF MS/MS) techniques. The obtained peptides were CRQNTLGHNTQTSIAQ (**14**) (C_70_H_118_N_26_O_26_S, 1,771 Da), EVSQGRP (**15**) (C_31_H_53_N_11_O_12_, 771 Da), VSRHFASYAN (**16**) (C_51_H_74_N_16_O_15_, 1,151 Da), and SAAVGSP (**17**) (C_24_H_41_N_7_O_10_, 587 Da). All four peptides exhibit potent ACE inhibitory activities, with IC_50_ values of 0.05, 0.08, 0.21, and 1.71 mmol.L^–1^, respectively (Forghani et al., 2016).Commercial composite proteinases were used to isolate and identify three biologically active peptides with ACE inhibitory activity from the gonads of *S. japonicus*. The peptides obtained were EIYR (**11**) (C_26_H_41_N_7_O_8_, 579 Da), LF (**12**) (C_15_H_22_N_2_O_3_, 278 Da), and NAPHMR (**13**) (C_29_H_48_N_12_O_8_S, 724 Da) (Zhong et al., 2018). NAPHMR exhibits the highest ACE inhibitory activity, which can be attributed to its specific amino acid residues. The Arg, His, and Asn residues can form hydrogen or π bonds with the S2 pocket or Zn^2+^-binding sequence of ACE, resulting in ACE inhibitory activity (Zhong et al., 2018).

### 2.3 Metal ion chelation

Despite their low concentrations in the body, metal ions play a significant role in maintaining health. They are involved in the metabolism of enzymes, hormones, vitamins, and nucleic acids. Their physiological functions primarily involve providing essential elements to body tissues or organs (Daengprok et al., 2003), serving as enzyme components or activators (Bozalioglu et al., 2005), and regulating oxidative stress.

Zinc, one of the most important trace elements in the human body, is involved in the regulation of over 300 enzymes and has significant biological and structural functions in various cellular processes (Bozalioglu et al., 2005). Alkaline proteinase was used for the enzymatic hydrolysis of the body wall of *S. japonicus*. Various techniques, including ultrafiltration, ion-exchange chromatography, gel filtration chromatography, and ultra-performance liquid chromatograph quadrupole time-of-flight tandem mass spectrometry (UPLC-Q-TOF MS/MS), were later used to isolate, purify, and identify the sea cucumber octapeptide WLTPTYPE (**20**), which has a molecular formula of C_49_H_67_N_9_O_14_ and molecular weight of 1,006 Da (Liu et al., 2019b). WLTPTYPE binds to zinc and other polypeptides via carboxyl and acylamide ligands, thereby promoting zinc-ion absorption. Two other zinc-chelated peptides, AATGVMPLDM (**21**) (C_42_H_72_N_10_O_14_S_2_, 1,004 Da) and AAYCATKFA (**22**) (C_43_H_64_N_10_O_12_S, 944 Da), were eventually isolated and purified. All three cucumber peptides exhibit the potential to effectively chelate zinc, which may be related to interactions between their free carboxyl groups and zinc (Liu et al., 2019a).

Calcium is the most abundant inorganic element in the human body, accounting for approximately 1.5%–2.2% of the body’s weight (Daengprok et al., 2003). Calcium deficiency can lead to metabolic bone diseases, such as osteoporosis and rickets. Trypsin enzymolysis was used to produce sea cucumber ovum hydrolysates (SCOHs) from the sea cucumber ovum (Sun et al., 2017b). HPLC was then used to isolate and obtain two peptides from these SCOHs: the octapeptide EDLAALEK (**23**) (C_38_H_65_N_9_O_15_, 888 Da) (Cui et al., 2019a) and heptapeptide NDEELNK (**24**) (C_34_H_56_N_10_O_16_, 860 Da) (Cui et al., 2019b). Glutamic and aspartic acids at the C-terminus of the two peptides can specifically bind to calcium ions and significantly enhance the absorption of Ca^2+^ by human colon cancer Caco-2 cells during gastrointestinal digestion. This finding suggests that these peptides have the potential to be effective nanocarriers for Ca^2+^ transport through the gastrointestinal system (Cui et al., 2018).

Iron is a crucial constituent of metalloproteins and serves as a vital repair factor in the regulation, activation, and modulation of numerous enzyme-catalysed reactions (Kazemi-Taskooh and Varidi, 2021). Iron deficiency is associated with an increased risk of diseases such as iron deficiency-related anaemia and neurodegenerative diseases. Insufficient iron intake and utilisation are the primary causes of iron deficiency. Neutral proteases were used to enzymatically hydrolyse sea cucumbers and isolated and purified a novel iron-chelating peptide with a molecular weight of <5 kDa by using ultrafiltration and mass spectrometry techniques (Fan et al., 2023). This peptide exhibits high iron-chelating activity. Additionally, the resulting chelate exhibits strong free radical scavenging abilities and shows potential as an efficient iron supplement (Fan et al., 2023). The type of hydrolase and extent of hydrolysis can also significantly affect iron-binding capacities. For example, iron-chelating peptides have been produced by the enzymatic hydrolysis of the body wall of *S. japonicus* by alkaline proteases, which have molecular weights ranging from 200 to 1,000 Da and consist of hydrophilic amino acid residues, including aspartic acid, histidine, and arginine. The iron-binding capacity of the sea cucumber ovum hydrolysate reached its highest level (92.1%) when the degree of hydrolysis was 25.9% (Sun et al., 2017a).

### 2.4 Neuroprotection

The incidence of neurodegenerative diseases associated with cognitive decline and memory loss is increasing owing to the global ageing population. Neurodegenerative diseases manifest primarily as inflammation, cholinergic dysfunction, oxidative stress, and amyloid plaques (Wang et al., 2021). The focus of studies on neurodegenerative diseases has shifted toward the identification of safe and effective active substances that can alleviate these diseases.

The sea cucumber heptapeptide NDEELNK (**24**) was successfully isolated from SCOHs. The molecular formula of this peptide is C_34_H_56_N_10_O_16_, and its molecular weight is 860 Da (Cui et al., 2018). NDEELNK enhances the PKA/BDNF/NGF signalling pathway in rat adrenal pheochromocytoma PC12 cells by increasing acetylcholine (ACh) levels and acetylcholinesterase (AChE) activity (Zhao et al., 2021). The hexapeptide FYDWPK (**25**), derived from *A. japonicus*, mitigates cognitive and memory impairment by regulating oxidative imbalance, reducing cholinergic dysfunction, and alleviating pathological alterations. This hexapeptide has the molecular formula C_44_H_54_N_8_O_10_ and a molecular weight of 854 Da (Zhao et al., 2022). Similar studies by Lin et al. involved the production of sea cucumber peptides that could alleviate memory damage by the enzymolysis of *S. japonicas* with neutral proteases. Treatment with sea cucumber peptides improves the behaviour of mouse models in a dose-dependent manner, modulates the cholinergic system, and counteracts scopolamine-induced cognitive impairment by upregulating long-term potentiation pathways and unsaturated fatty acid levels (Lu et al., 2022). A potential connection between the memory-enhancing effects of sea cucumber peptides and the suppression of hippocampal histone acetyltransferase activity has been suggested (Xu et al., 2020). Further studies on molecular regulatory mechanisms of sea cucumber peptides have revealed the ability to exert neuroprotective effects through the regulation of post-transcriptional hippocampal protein acetylation (Lu et al., 2021b).

An effective approach to alleviate the neuropathological characteristics of Alzheimer’s disease is to inhibit the aggregation of amyloid beta (Aβ) peptides. In a previous study, *S. japonicas* was enzymatically hydrolysed using pepsin, followed by purification via gel permeation chromatography, anion exchange chromatography, size-exclusion chromatography, and electrospray ionization mass spectrometry (ESI-MS/MS). This process yielded IGFH (**26**) (C_23_H_32_N_6_O_5_, 472 Da), LGFH (**27**) (C_23_H_32_N_6_O_5_, 472 Da), DWF (**28**) (C_24_H_26_N_4_O_6_, 466 Da), and FQF (**29**) (C_23_H_28_N_4_O_5_, 440 Da). All four peptides demonstrate effective CD38 inhibition and anti-Aβ aggregation activities, suggesting a potential strategy for the prevention or treatment of Alzheimer’s disease (Lin et al., 2019).

### 2.5 Wound healing

Sea cucumbers exhibit robust visceral regeneration capabilities and are used in traditional Asian medicine for wound healing. Pilus et al. synthesised a short 45-amino acid peptide, Sh-EGFl-1, with a molecular weight of approximately 4.9 kDa using the transcriptome database of *S. horrens*. Sh-EGFl-1 demonstrates strong molecular interactions with human epidermal growth factor receptor (hEGFR), suggesting its potential to enhance cell proliferation. Its mechanisms of action may involve the activation of the PI3K, MAPK, PLC-γ, JAK-STAT, and Rho pathways (Pilus et al., 2022).

Small-molecule active peptides have significant potential in promoting wound healing. Sea cucumber tetrapeptides containing the VLLY (**30**) (C_26_H_42_N_4_O_6_, 506 Da) and VTPY (**31**) (C_23_H_34_N_4_O_7_, 479 Da) sequences have been found to effectively promote the proliferation and migration of human skin fibroblasts and human umbilical vein endothelial cells. These peptides also enhance wound healing by increasing mitochondrial respiration and upregulating the ERK/AKT pathway by inhibiting the binding of MKP to ERK2 and PHLPP to AKT (Zheng et al., 2022). Small molecule oligopeptides from sea cucumbers (SCCOPs) were further purified using *Codonopsis pilosula* enzymatic hydrolysates with a molecular weight of <1 kDa. Treatment with these SCCOPs results in favourable outcomes in the inflammatory response, vascularisation, collagen deposition, oxidative stress, and nutritional status, thereby significantly promoting wound healing in a *db/db* diabetic mouse model (Li et al., 2018a). Subsequent investigations revealed that sea cucumber collagen oligopeptides prepared using the same method effectively stimulate NO generation and neovascularisation, reduce the inflammatory response, and enhance wound tensile strength at the wound sites of db/db diabetic mice, thereby facilitating the healing process (Li et al., 2017a).

### 2.6 Antihyperuricaemic activity

Hyperuricaemia is the second most common metabolic disorder in humans and is characterised by a persistent elevation in serum uric acid levels. Trypsin and alkaline proteases were used in a 2:1 ratio to enzymatically hydrolyse *A. japonicus* and *A. leucoprocta* to produce two peptides, the enzymatic hydrolysates of *A. japonicus* (EH-JAP) and *A. leucoprocta* (EH-LEU), with antihyperuricaemic activity. Each peptide has a molecular weight of <1 kDa (Wan et al., 2020). Both EH-JAP and EH-LEU can alleviate hyperuricaemia and kidney inflammation in mice with diet-induced hyperuricaemia, possibly by inhibiting the TLR4/MyD88/NF-κB signalling pathway, thereby inhibiting uric acid biosynthesis and promoting its excretion (Wan et al., 2020). Under identical conditions, EH-JAP was enzymatically hydrolysed and purified to yield two novel sea cucumber hexapeptides: GPAGPR (**32**) (C_23_H_39_N_9_O_7_, 553 Da) and GPSGRP (**33**) (C_23_H_39_N_9_O_8_, 569 Da). These peptides were found to exhibit antihyperuricaemic activity by inhibiting uric acid biosynthesis and reabsorption. The underlying molecular mechanisms may involve the regulation of the gut microbiota and host miRNA (Fan et al., 2022).

### 2.7 Antitumor effects

Increases in the population, ageing, and lifestyle-related risk factors are expected to lead to significant increases in the incidence of malignant tumours and associated mortality rates (Torre et al., 2016). Sea cucumbers contain a diverse range of amino acids, especially essential amino acids, which have the potential to impede tumour progression.

Breast cancer is a predominant malignancy among women worldwide. Sea cucumber intestinal peptides (SCIPs) can be derived from the alkaline protease hydrolysis of the *Atlantic sea cucumber* intestinal tract. An analysis of the amino acid composition of SCIPs revealed the predominant presence of hydrophobic and branched-chain amino acids with molecular weights <1 kDa. SCIPs can effectively inhibit the proliferation of MCF-7 breast cancer cells and induce apoptosis in zebrafish, possibly through the inhibition of the PI3K/AKT signalling pathway (Wei et al., 2021). Two peptides, WPPNYQW (**34**) (C_50_H_59_N_11_O_11_, 989 Da) and YDWRF (**35**) (C_39_H_47_N_9_O_9_, 785 Da), were identified in *C. frondose* (Zhang et al., 2020). A molecular docking analysis using AutoDock Vina software demonstrated the stable binding of WPPNYQW to the active sites of overexpressed EGFR, PI3K, AKT1, and CDK4 proteins in breast cancer. Similarly, YDWRF stably binds to the active sites of PI3K and AKT1. These findings suggest the potential of both WPPNYQW and YDWRF as inhibitors of breast cancer (Wargasetia et al., 2021).

Lung cancer is currently the most lethal malignant tumour in humans, with non-small cell lung cancer accounting for approximately 85% of all lung cancers and exhibiting a survival rate of only 15% (Molina et al., 2008; Iqbal et al., 2019). Sea cucumber peptides with anti-tumour efficacy can be obtained through the enzymatic hydrolysis of *Russian polar ginseng* using a combination of papain and trypsin in a 2:1 ratio. *In vitro* and *in vivo* experiments have revealed that these peptides inhibit the proliferation, migration, and invasion of A549 non-small cell lung cancer cells, formation of pleural effusion, and tumour growth in mice with lung cancer. Additionally, they alleviate liver and kidney damage and prolong the survival time of mice; the underlying molecular mechanisms may involve the regulation of miR-378a-5p, which targets the tumour suppressor TUSC2 (Mao et al., 2021).

### 2.8 Anti-fatigue properties

Anti-fatigue peptides have gained significant attention in recent studies because of their potential health benefits, such as high biological activity, low molecular weights, easy absorption, and low toxicity (Zhao et al., 2016). Anti-fatigue peptides rich in glycine, glutamic acid, and proline from *A. japonicus* were prepared using a neutral protease with a molecular weight of <2 kDa. The mechanism of action of these peptides may involve the regulation of energy metabolism, reduction of oxidative damage and inflammation, and downregulation of TRL4 and NF-κB expression (Ye et al., 2017). Previous studies have identified such peptides with molecular weights of <5 kDa. Treatment with sea cucumber peptides has been found to significantly increase power-sensitive factors, lipid decomposition metabolism, and mRNA and protein levels of mitochondrial biogenesis factors in C57BL/6J mice. In addition, gluconeogenic mRNA levels are significantly upregulated. This anti-fatigue effect may be attributed to the enhancement of mitochondrial function through energy regulation and antioxidative improvement (Yu et al., 2020). A composite protease preparation was used to enzymatically hydrolyse *A. japonicus*, which resulted in the production of sea cucumber oligopeptides with molecular weights <1 kDa. These peptides could effectively reduce serum urea nitrogen content, improve glycogen storage capacity *in vivo*, significantly prolong the swimming endurance of weight-carrying mice, and relieve fatigue (Wang et al., 2022).

### 2.9 Hypoglycaemic activity

Diabetes is a complex metabolic disorder characterised by elevated blood sugar levels and persistent hyperglycaemia. Prolonged metabolic syndrome can result in chronic damage and dysfunction in multiple organs. In a previous study, the gastric and gastrointestinal digestion fractions of *A. japonicus* were obtained by simulating gastrointestinal tract digestion. Administration of both hydrolysates improved glucose uptake in 3T3-L1 and insulin-resistant HepG2 cells induced by high insulin levels. The <3 kDa fraction showed the strongest inhibitory activity against dipeptidyl peptidase IV (Gong et al., 2020). Diabetic nephropathy is a significant complication of diabetes. Peptides with a molecular weight <3 kDa were derived from *A. japonicus* through enzymatic hydrolysis using pancreatic proteases and purification via matrix-assisted laser desorption/ionisation time-of-flight mass spectrometry and other techniques. These peptides were found to inhibit the MAPK and p38 MAPK signalling pathways, thereby influencing glucose and lipid metabolism and exerting regulatory effects on the occurrence and progression of diabetic nephropathy (Dong et al., 2018). Li et al. conducted a similar study and obtained sea cucumber peptide fractions (PFs) using a composite protease hydrolysis method. The PFs effectively reduced urinary glucose and urea levels and exerted hypoglycaemic and renoprotective effects in *db/db* mice (Li et al., 2017b).

### 2.10 Promotion of bone growth

Osteoblasts play a “builder” role in bone growth and maintaining internal equilibrium. Increasing the number of osteoblasts has consistently been an important objective in bone health research. Marine source-derived peptides have demonstrated favourable osteogenic properties. The enzymatic hydrolysis of sea cucumber intestines using alkaline proteases and intestinal self-digesting enzymes has been found to allow the extraction of SCIPs. These peptides are rich in glutamic acid/glutamine and have a molecular weight ranging from 200 to 1,000 Da and purity of 68.8% (Yue et al., 2022a). SCIPs exhibit osteogenic activity and enhance cell cycle progression by upregulating histone acetylation via glutamine-mediated mechanisms. These effects promote the proliferation of growth plate chondrocytes, thereby promoting longitudinal bone growth (Yue et al., 2022a). Further use of activity-tracking techniques to analyse sea cucumber intestinal hydrolysates has led to the discovery of 28 novel bone-forming peptides. Among these peptides, some with a molecular weight <3 kDa exhibit promising integrin-binding capabilities. Such binding has the potential to enhance the differentiation of growth plate chondrocytes into osteoblasts through integrin-mediated mechanisms, thereby facilitating bone formation (Yue et al., 2022b).

### 2.11 Promotion of collagen synthesis and secretion

To delay skin ageing, growing emphasis has been placed on understanding skin ageing and developing methods to enhance fibroblast proliferation and collagen expression. A composite enzyme consisting of alkaline protease, papain, and pancreatin in a 3:3:4 ratio was used to hydrolyse the body wall of *S. japonicus*. An active tracking method using techniques such as ion-exchange and gel column chromatography was further used to isolate a sea cucumber peptide, SP12, with a molecular weight of approximately 3.87 kDa. This peptide effectively promotes NIH/3T3 embryonic fibroblast proliferation and collagen secretion (Song et al., 2017). At a concentration of 200 g mL^−1^, the proliferation of NIH/3T3 cells and secretion of collagen proteins are enhanced; however, when SP12 concentration is increased to 800 g mL^−1^, NIH/3T3 cell proliferation is inhibited, whereas collagen protein secretion is significantly enhanced. SP12 has a more significant effect on promoting NIH/3T3 cell proliferation than commonly used antiwrinkle ingredients, such as oat peptides and snail secretion filtrates (Chai et al., 2015). Similar studies have shown that peptides derived from *A. leucoproata* through enzymatic hydrolysis using neutral and animal proteases at a mass ratio of 3:2 can promote the proliferation of human skin fibroblasts and stimulate collagen synthesis. Notably, peptides with a molecular weight <3 kDa have been found to exhibit the strongest collagen secretion ability (Jiang et al., 2014).

### 2.12 Anti-inflammation

Inflammation is a typical reaction to infection; however, an excessive inflammatory response can exacerbate autoimmune or autoimmune inflammatory disorders. An anti-inflammatory peptide with a molecular weight ranging from 180 to 1,000 Da and purity of 72.12% was previously derived from the enzymatic hydrolysis of sea cucumber using flavourzyme. It effectively inhibits a lipopolysaccharide-induced inflammatory response and upregulates HO-1 mRNA expression in RAW264.7 macrophages (Song et al., 2016). Sea cucumber peptides also affect the recruitment of inflammation-related leucocytes. Hydrolysates derived from the enzymatic hydrolysis of *A. japonicus* have been found to significantly block the migration of white blood cells to the site of injury and effectively mitigate CuSO_4_-induced neuroinflammatory damage in zebrafish (Zhang et al., 2021).

### 2.13 Immunological regulation

Natural-source nutritional interventions have emerged as effective methods for improving immunological function and disease resistance. HPLC and matrix-assisted laser desorption ionisation timeof-flight mass spectrometry (MALDI-TOF-MS) techniques were used to isolate and purify small molecule oligopeptides from sea cucumbers (SOPs) (<1 kDa). These SOPs were obtained from Codonopsis pilosula enzyme hydrolysates. SOPs can induce both innate and adaptive immune responses. Specifically, they can increase macrophage phagocytosis and natural killer cell activity and promote cell-mediated and humoral immunity (He et al., 2016). In addition, sea cucumber peptides can modulate the diversity of intestinal flora and upregulate the immune response of T lymphocyte subsets. These findings suggest that they may have antiallergic properties, as demonstrated in a mouse model of ovalbumin allergy, and may exhibit effective immune regulation abilities (Yun et al., 2022).

## 3 Discussion

Low-molecular-weight sea cucumber peptides are gaining increasing attention owing to their advantages in biological absorption compared with sea cucumber proteins. Many studies have focused on reducing the molecular weights of sea cucumber hydrolysates to specific thresholds, such as <1, <3, <5, or <10 kDa, to enhance their absorption and utilisation. The choice of enzyme is crucial for the enzymatic hydrolysis of sea cucumber peptides (Auwal et al., 2019; Gong et al., 2020; Fan et al., 2023). Enzymes commonly employed in various applications include alkaline protease, neutral protease, pancreatic protease, flavour protease, bromelain, papain, and pepsin. Sea cucumber peptides with various biological activities can be generated by modifying factors such as the enzymatic hydrolysis temperature, pH, and duration. In addition, the combination of different hydrolytic enzymes and microwave-assisted enzymatic hydrolysis may be a viable approach for enzymatic hydrolysis. The extraction, separation, and purification of sea cucumber peptides are crucial for improving their absorption and utilisation rates. Various techniques, including ultrafiltration, gel filtration chromatography, ion-exchange chromatography, size-exclusion chromatography, RP-HPLC, and LS-MS/MS, can be employed to isolate and purify sea cucumber peptides and obtain high-purity bioactive peptides.

**FIGURE 4:**
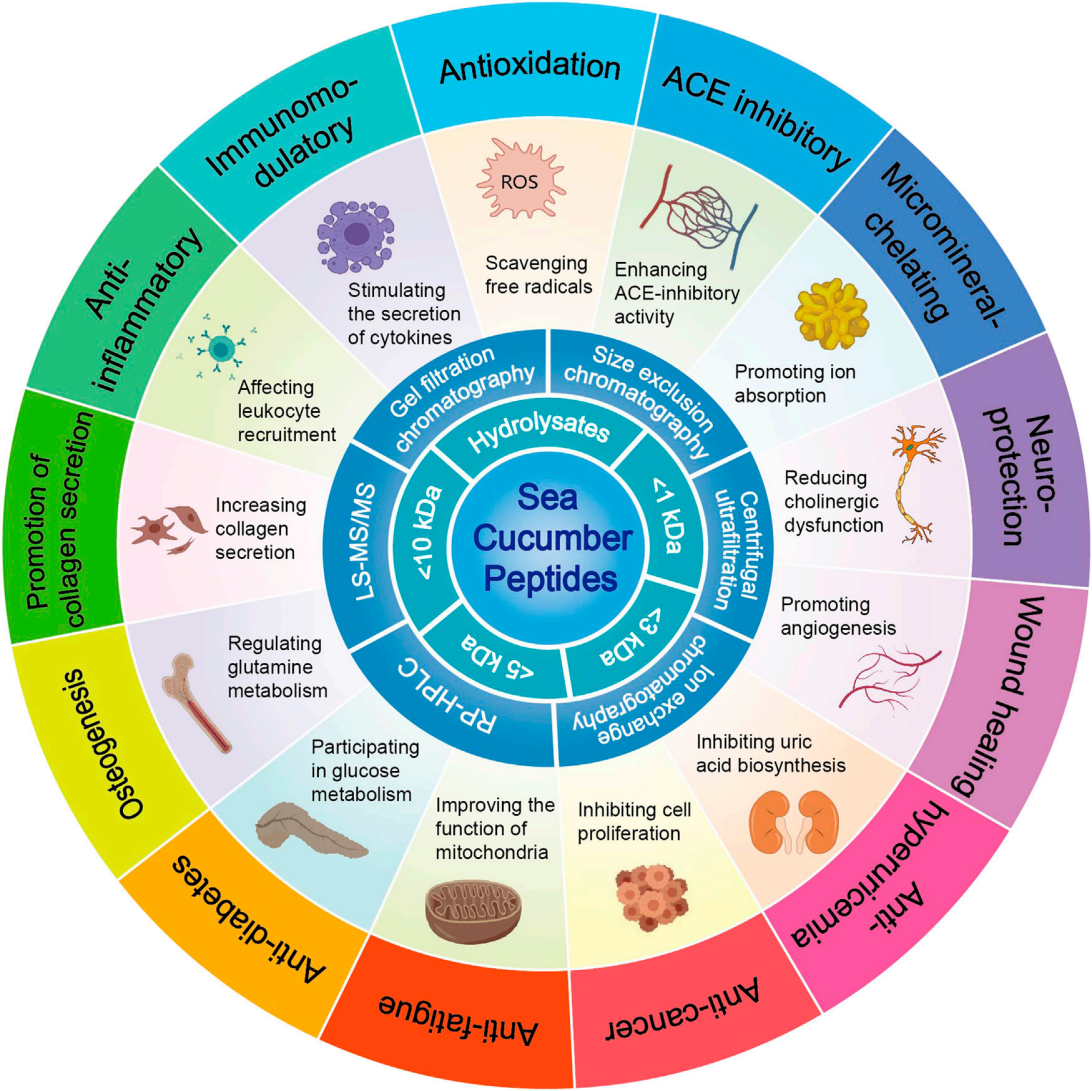
Biological activity of sea cucumber polypeptides.

The currently known biological activities of sea cucumber peptides include antioxidative, blood pressure-lowering, metal ion-chelating, neuroprotective, wound healing, anti-hyperuricaemic, anti-tumour, anti-fatigue, blood sugar-lowering, bone growth-promoting, collagen-promoting, anti-inflammatory, and immune-regulating activities (Figure 4). Despite these known activities, our understanding of the structural characteristics and structure–activity relationships of small-molecule sea cucumber active peptides remains limited. This knowledge gap suggests a potential avenue for future research on sea cucumber peptides. An increasing number of studies highlight the unique efficacy of sea cucumber peptides in inhibiting the occurrence, invasion, and metastasis of tumour cells by improving tumour inflammation and enhancing immune function, thereby suppressing the further development of tumours. Therefore, these peptides offer novel targeted strategies for tumour treatment (Mao et al., 2021; Wei et al., 2021). Furthermore, the collagen-promoting and antioxidative properties of sea cucumber peptides highlight their use as promising ingredients in the cosmetics industry as they offer several advantages, including skin texture improvement, skin barrier repair, and antioxidant benefits. Therefore, sea cucumbers hold potential as valuable raw materials in the cosmetics sector.
