# Supplementary material for: Research progress on the chemical components and biological activities of sea cucumber polypeptides
Source: Front Pharmacol. 2023 Oct 16;14:1290175. doi: 10.3389/fphar.2023.1290175 (PMC10613643; doi:10.3389/fphar.2023.1290175)
Supplement: Supplementary file 1 [file Table1.docx]

**Supplementary Table 1.** Molecular formula, preparation process, species, and molecular mechanism of different bioactive sea cucumber peptides

| peptides | Molecular formula | Amino acid formula | MW | Structural formula | Structural characteristics | Purity | Enzymatic hydrolysis process | Purification and identification process | species | Molecular Mechanism | references |
| --- | --- | --- | --- | --- | --- | --- | --- | --- | --- | --- | --- |
| **Antioxidant Activity** | | | | | | | | | | | |
| FETLMPLWGNK | C_63_H_94_N_14_O_16_S | Phe-Glu-Thr-Leu-Met-Pro-Leu-Trp-Gly-Asn-Lys | 1 336 Da | (**1**) | / | >95% | Trypsin(0.12%, pH = 8, 45℃, 4 h)→papain(0.05%, pH = 6, 60℃, 4 h) | Ultrafiltration(<3 kDa)； Size exclusion chromatography；LC-MS/MS analysis | *Apostichopus japonicus* | Compensating for glutathione depletion, decreasing mitoc hondrial superoxide levels, and protecting mitochondria from superoxide-induced mitophagy. | (Lu et al., 2021a) |
| HEPFYGNEGALR | C_62_H_88_N_18_O_19_ | His-Glu-Pro-Phe-Tyr-Gly-Asn-Glu-Gly-Ala-Leu-Arg | 1 389 Da | (**2**） | / | >95% | Trypsin(0.12%, pH = 8, 45℃, 4 h)→papain(0.05%, pH = 6, 60℃, 4 h) | Ultrafiltration(<3 kDa）； Size exclusion chromatography；LC-MS/MS analysis | *Apostichopus japonicus* | Activating the Nrf2/HO-1 pathway and blocking the nuclear translocation of NF-κB | (Lu et al., 2021a; Zhu et al., 2022) |
| KMYPVPLN | C_45_H_72_N_10_O_11_S | Lys-Met-Tyr-Pro-Val-Pro-Leu-Asn | 961 Da | (**3**) | / | >95% | Trypsin(0.12%, pH = 8, 45℃, 4 h)→papain(0.05%, pH = 6, 60℃, 4 h) | Ultrafiltration(<3 kDa)； Size exclusion chromatography；LC-MS/MS analysis | *Apostichopus japonicus* | Compensating for glutathione depletion, decreasing mitochondrial superoxide levels, and protecting mitochondria from superoxide-induced  mitophagy. | (Lu et al., 2021a) |
| *FLAP | C_23_H_34_N_4_O_5_ | Phe-Leu- Ala-Pro | 446 Da | (**4**） | hydrophobicity | / | Neutrase(5%, pH = 7, 55℃, under irradiation 300 W, 0.5 h) | Ultrafiltration(<1 kDa)；HPLC(TSKGel 2 000 SWXL)；Gel Filtration Chromatography(Sephadex G-15 gel column)；RP-HPLC(Zorbax SB-C18)；N-terminal amino acid sequencing | *Acaudina molpadioides* | Protecting cells from oxidative damage through the JNK/Nrf2 pathway | (Jin et al., 2019; Jin et al., 2021) |
| CPs | / | / | ≤ 1 kDa | / | / | / | Alcalase(under irradiation 250 W, 0.5 h, ≥200 U.mg-1, pH = 10, 45℃, 1 h) | Ultrafiltration(<1 kDa) | *Acaudina molpadioides* | Decreasing the level of ROS and MDA and increasing the activity of antioxidant enzymes | (Li et al., 2019) |
| A_<2_ | / | / | 636-1 388 Da | / | c-terminal amino acid： Tyr, Trp, Phe, Leu, Val, Pro and Lys. | / | Alcalase(25℃, pH = 7.5, 2 h) | Vacuum filtration(0.22 µm membrane)；ultrafiltration；UPLC；LC-MS/MS analysis | *Cucumaria frondosa* | / | (Zhang et al., 2020) |
| T_<2_ | / | / | 698-2 286 Da | / | c-terminal amino acid： Arg or Lys | / | Trypsin(25℃, pH = 7.5, 2 h) | Vacuum filtration(0.22 µm membrane)；ultrafiltration；UPLC；LC-MS/MS analysis | *Cucumaria frondosa* | / | (Zhang et al., 2020) |
| sea cucumber peptides | / | / | <3 kDa | / | / | / | Autolytic enzyme(E/S = 1:1, pH = 6, 60℃, 24 h) | Ultrafiltration(<3 kDa)；semi-preparative HPLC(80 x 10 mm Hi-Pore Reversed Phase Column) | *Isostichopus fuscus* | / | (Hernández-Sámano et al., 2015) |
| sea cucumber peptides | / | / | <10 kDa | / | rich in Gly and Ala | / | Neutrase(1 800 u.g^-1^, pH = 7.5, 55℃, 3 h) | Ultrafiltration(<10 kDa) | *Holothuriacoluber Semper；Acaudinamobpadioides Semper；Apostichopus japonicus；Parastichopus californicus* | / | (Zhao et al., 2012) |
| sea cucumber peptides | / | / | / | / | / | / | Autolytic enzyme (under Ultraviolet irradiation for 25 min, pH = 7.5, 4 h) | Ultrafiltration: SPH I (>10 kDa), SPH II (5-10 kDa), SPH III (3-5 kDa), SPH IV (<3 kDa) | *Stichopus japonicus* | / | (Wang et al., 2010) |
| **Lowering Blood Pressure** | | | | | | | | | | | |
| *ALGPQFY | C_39_H_54_N_8_O_10_ | Ala-Leu-Gly-Pro-Gln-Phe-Tyr | 794 Da | (**5**) | / | > 90% | Bromelain(2%, pH = 7, 40℃, 4 h) | Ultrafiltration(<10 kDa); semi preparative RP-HPLC(C18); LC-QTOF | *Actinopyga*  *lecanora* | Enhancing ACE-inhibitory activity | (Auwal et al., 2019) |
| *KVPPKA | C_30_H_54_N_8_O_7_ | Lys-Val-Pro-Pro-Lys-Ala | 639 Da | (**6**) | / | > 90% | Bromelain(2%, pH = 7, 40℃, 4 h) | Ultrafiltration(<10 kDa); semi preparative RP-HPLC(C18); LC-QTOF | *Actinopyga*  *lecanora* | Enhancing ACE-inhibitory activity | (Auwal et al., 2019) |
| *LAPPTM | C_29_H_52_N_6_O_8_S | Leu-Ala-Pro-Pro-Thr-Met | 629 Da | (**7**) | / | > 90% | Bromelain(2%, pH = 7, 40℃, 4 h) | Ultrafiltration(<10 kDa); semi preparative RP-HPLC(C18); LC-QTOF | *Actinopyga*  *lecanora* | Enhancing ACE-inhibitory activity | (Auwal et al., 2019) |
| *EVLIQ | C_27_H_48_N_6_O_9_ | Glu-Val-Leu-Ile-Gln | 601 Da | (**8**) | / | > 90% | Bromelain(2%, pH = 7, 40℃, 4 h) | Ultrafiltration(<10 kDa); semi preparative RP-HPLC(C18); LC-QTOF | *Actinopyga*  *lecanora* | Enhancing ACE-inhibitory activity | (Auwal et al., 2019) |
| *EHPVL | C_27_H_43_N_7_O_8_ | Glu-His-Pro-Val-Leu | 594 Da | (**9**) | / | > 90% | Bromelain(2%, pH = 7, 40℃, 4 h) | Ultrafiltration(<10 kDa); semi preparative RP-HPLC(C18); LC-QTOF | *Actinopyga*  *lecanora* | Enhancing ACE-inhibitory activity | (Auwal et al., 2019) |
| MEGAQEAQGD | C_39_H_62_N_12_O_19_S | Met-Glu-Gly-Ala-Gln-Glu-Ala-Gln-Gly-Asp | 1 034 Da | (**10**) | / | 0.55% | Bromelain(1%, pH = 7, 45℃, 3 h)→Alcalase(2%, pH = 7.5, 55℃, 3 h) | Ultrafiltration(<2 kDa); ion-exchange chromatography; RP-HPLC(Zorbax C18); NanoESI-Q-TOF MS/MS | *Acaudina molpadioides* | Enhancing ACE-inhibitory activity | (Zhao et al., 2009) |
| EIYR | C_26_H_41_N_7_O_8_ | Glu-Ile-Tyr-Arg | 579 Da | (**11**) | / | / | Protamex(0.75%, pH = 6.5, 45℃, 1.95 h) | MW cutoff membranes(<3 kDa); RP-HPLC(Sephadex G-15 gel column); MALDI-TOF-MS/MS | *Stichopus japonicus* | Enhancing ACE-inhibitory activity | (Zhong et al., 2018) |
| LF | C_15_H_22_N_2_O_3_ | Leu-Phe | 278 Da | (**12**) | / | / | Protamex(0.75%, pH = 6.5, 45℃, 1.95 h) | MW cutoff membranes(<3 kDa); RP-HPLC(Sephadex G-15 gel column); MALDI-TOF-MS/MS | *Stichopus japonicus* | Enhancing ACE-inhibitory activity | (Zhong et al., 2018) |
| NAPHMR | C_29_H_48_N_12_O_8_S | Asn-Ala-Pro-His-Met-Arg | 724 Da | (**13**) | / | 98.9% | Protamex(0.75%, pH = 6.5, 45℃, 1.95 h) | MW cutoff membranes(<3 kDa); RP-HPLC(Sephadex G-15 gel column); MALDI-TOF-MS/MS | *Stichopus japonicus* | Enhancing ACE-inhibitory activity | (Zhong et al., 2018) |
|  |  |  |  |  |  |  |  |  |  |  |  |
| CRQNTLGHNTQTSIAQ | C_70_H_118_N_26_O_26_S | Cys-Arg-Gln-Asn-Thr-Leu-Gly-His-Asn-Thr-Gln-Thr-Ser-Ile-Ala-Gln | 1 771 Da | (**14**) | / | > 97% | Alcalase(2 103 U.mL^-1^, pH = 7.5, 55°C, 5 h) | Ultrafiltration(<10 kDa); RP-HPLC(Semiprep ZORBAX 300SB C18); isoelectric focusing electrophoresis；ESI-Q-TOF MS/MS | *Stichopus horrens* | Inhibiting ACE in a non-competitive manner | (Forghani et al., 2016) |
| EVSQGRP | C_31_H_53_N_11_O_12_ | Glu-Val-Ser-Gln-Gly-Arg-Pro | 771 Da | (**15)** | / | > 97% | Alcalase(2 103 U.mL^-1^, pH = 7.5, 55°C, 5 h) | Ultrafiltration(<10 kDa); RP-HPLC(Semiprep ZORBAX 300SB C18); isoelectric focusing electrophoresis；ESI-Q-TOF MS/MS | *Stichopus horrens* | Exhibiting mixed inhibition patterns to degradation by ACE | (Forghani et al., 2016) |
| VSRHFASYAN | C_51_H_74_N_16_O_15_ | Val-Ser-Arg-His-Phe-Ala-Ser-Tyr-Ala-Asn | 1 151 Da | (**16**) | / | > 97% | Alcalase(2 103 U.mL^-1^, pH = 7.5, 55°C, 5 h) | Ultrafiltration(<10 kDa); RP-HPLC(Semiprep ZORBAX 300SB C18); isoelectric focusing electrophoresis；ESI-Q-TOF MS/MS | *Stichopus horrens* | Exhibiting mixed inhibition patterns to degradation by ACE | (Forghani et al., 2016) |
| SAAVGSP | C_24_H_41_N_7_O_10_ | Ser-Ala-Ala-Val-Gly-Ser-Pro | 587 Da | (**17**) | / | > 97% | Alcalase(2 103 U.mL^-1^, pH = 7.5, 55°C, 5 h) | Ultrafiltration(<10 kDa); RP-HPLC(Semiprep ZORBAX 300SB C18); isoelectric focusing electrophoresis；ESI-Q-TOF MS/MS | *Stichopus horrens* | Exhibiting mixed inhibition patterns to degradation by ACE | (Forghani et al., 2016) |
| *PNVA | C_17_H_29_N_5_O_6_ | Pro-Asn-Val-Ala | 399 Da | (**18**) | / | / | Trypsin and papain (2.5 kU.g^-1^, pH = 7, 50°C, 4 h) | Chromatographic fractionation(Sephadex G-15 gel column); RP-HPLC(Zorbax SB-C18); MALDI-TOF MS/MS | *Acaudina molpadioidea* | Enhancing ACE-inhibitory activity | (Li et al., 2018b) |
| *PNLG | C_17_H_29_N_5_O_6_ | Pro-Asn-Leu-Gly | 399 Da | (**19**) | / | / | Trypsin and papain (2.5 kU.g^-1^, pH = 7, 50°C, 4 h) | Chromatographic fractionation(Sephadex G-15 gel column); RP-HPLC(Zorbax SB-C18); MALDI-TOF MS/MS | *Acaudina molpadioidea* | Enhancing ACE-inhibitory activity | (Li et al., 2018b) |
| sea cucumber peptides | / | / | / | / | rich in Gly, Glu, and Asp | / | Alcalase(E/S = 1：100, pH = 8, 37°C, 24 h) | O-phthaldialdehyde method (OPA); Khan method | *Actinopyga lecanora* | Enhancing ACE-inhibitory and anti-oxidative activities | (Ghanbari et al., 2015) |
| **Metal Ion Chelation** | | | | | | | | | | | |
| WLTPTYPE | C_49_H_67_N_9_O_14_ | Trp-Leu-Thr-Pro-Thr-Tyr-Pro-Glu | 1 006 Da | (**20**) | Pro and Leu | / | Alcalase(0.06%, pH = 8.5, 50℃, 5 h) | Ultrafiltration(<5 kDa); anion-exchange chromatography；gel filtration chromatography(Sephadex G-15 gel column); UPLC-Q-TOF-MS/MS(Zorbax SB-C18) | *Stichopus japonicus* | Binding zinc and peptides through carboxyl groups and amide ligands. | (Liu et al., 2019b) |
| AATGVMPLDM | C_42_H_72_N_10_O_14_S_2_ | Ala-Ala-Thr-Gly-Val-Met-Pro-Leu-Asp-Met | 1 004 Da | (**21**) | / | / | / | / | *Stichopus japonicus* | Binding zinc with free carboxyl in the C-terminus of peptides. | (Liu et al., 2019a) |
| AAYCATKFA | C_43_H_64_N_10_O_12_S | Ala-Ala-Tyr-Cys-Ala-Thr-Lys-Phe-Ala | 944 Da | (**22**) | / | / | / | / | *Stichopus japonicus* | Binding zinc with free carboxyl in the C-terminus of peptides. | (Liu et al., 2019a) |
| EDLAALEK | C_38_H_65_N_9_O_15_ | Glu-Asp-Leu-Ala-Ala-Leu-Glu-Lys | 888 Da | (**23**) | Glu and Asp | 98% | Trypsin(3 kU.g^-1^ , pH = 8, 37℃, 3 h) | HPLC(Superdex Peptide 10/300GL) | */* | Bounding to two carboxyl oxygen atoms of C-terminal Glu and Asp | (Cui et al., 2019a) |
| NDEELNK | C_34_H_56_N_10_O_16_ | Asn-Asp-Glu-Glu-Leu-Asn-Lys | 860 Da | (**24**) | Glu and Asp | 98.72% | Trypsin(3 kU.g^-1^, pH = 8, 37℃, 3 h) | RP-HPLC(C18 semi-preparative column); Nano-LC-ESI-MS/MS | */* | Bounding to two carboxyl oxygen atoms of C-terminal Glu and Asp | (Cui et al., 2019b) |
| sea cucumber peptides | / | / | <5 kDa | / | rich in Asp、Glu, Gly, and Pro | / | Neutral protease(100 U.mg^-1^, 50℃, 5 h) | Ultrafiltration (5 KDa); Mass spectrometry(RP-C18) | */* | Getting Stronger radical scavenging ability and potential high-efficiency iron sup- plementation ability | (Fan et al., 2023) |
| sea cucumber ovum hydrolysates (SCOHs) | / | / | 200-1 000Da | / | hydrophile ：Asp, Arg, and His | 54.0% | Alcalase (3 kU.g^-1^, pH = 8.5, 50℃, 3 h) | Gel-permeation chromatography(Superdex Pep-tide 10/300 GL column); HPLC; UPLC(Phenomenex Luna C18 column) | *Stichopus japonicus* | Bounding iron to the SCOHs through interactions with carboxyl oxygen of Asp, guanidine nitrogen of Arg, or nitrogen atoms in imi-dazole group of His | (Sun et al., 2017a) |
| **Neuroprotection** | | | | | | | | | | | |
| FYDWPK | C_44_H_54_N_8_O_10_ | Phe−Tyr−Asp−Trp−Pro−Lys | 854 Da | (**25**) | / | / | / | / | *Stichopus japonicas* | Regulating oxidative imbalance, reducing cholinergic dysfunction and relieving pathological alterations | (Zhao et al., 2022) |
| *NDEELNK | C_34_H_56_N_10_O_16_ | Asn-Asp-Glu-Glu-Leu-Asn-Lys | 860 Da | (**24**) | / | 98.72% | Trypsin(3 kU.g^-1^ , pH = 8, 37℃, 3 h) | UPLC(Phenomenex Luna C18 column); QTOF-MS/MS Analysis | / | Enhancing energy metabolism and upregulation of the PKA/BDNF/NGF signaling pathway | (Zhao et al., 2021) |
| IGFH | C_23_H_32_N_6_O_5_ | Ile-Gly-Phe-His | 472 Da | (**26**) | / | / | Pepsin(pH = 2, 37℃, 2 h)→Pancreatin(4%, pH = 2, 37℃, 2 h) | Gel permeation chromatography (Sephadex G-50 column); Anion-exchange chromatography(filled with DEAE-52 cellulose resins); Gel permeation chromatography (Sephadex G-25 column); Size exclusion chromatography(TSK G2000 SWXL column); ESI-MS/MS | *Stichopus japonicus* | Enhancing CD38 inhibitory activity and anti-Aβ aggregation activity | (Lin et al., 2019) |
| LGFH | C_23_H_32_N_6_O_5_ | Leu-Gly-Phe-His | 472 Da | (**27**) | / | / | Pepsin(pH = 2, 37℃, 2 h)→Pancreatin(4%, pH = 2, 37℃, 2 h) | Gel permeation chromatography (Sephadex G-50 column); Anion-exchange chromatography(filled with DEAE-52 cellulose resins); Gel permeation chromatography (Sephadex G-25 column); Size exclusion chromatography(TSK G2000 SWXL column); ESI-MS/MS | *Stichopus japonicus* | Enhancing CD38 inhibitory activity and anti-Aβ aggregation activity | (Lin et al., 2019) |
| DWF | C_24_H_26_N_4_O_6_ | Asp-Trp-Phe | 466 Da | (**28**) | / | / | Pepsin(pH = 2, 37℃, 2 h)→Pancreatin(4%, pH = 2, 37℃, 2 h) | Gel permeation chromatography (Sephadex G-50 column); Anion-exchange chromatography(filled with DEAE-52 cellulose resins); Gel permeation chromatography (Sephadex G-25 column); Size exclusion chromatography(TSK G2000 SWXL column); ESI-MS/MS | *Stichopus japonicus* | Enhancing CD38 inhibitory activity and anti-Aβ aggregation activity | (Lin et al., 2019) |
| FQF | C_23_H_28_N_4_O_5_ | Phe-Gln-Phe | 440 Da | (**29**) | / | / | Pepsin(pH = 2, 37℃, 2 h)→Pancreatin(4%, pH = 2, 37℃, 2 h) | Gel permeation chromatography (Sephadex G-50 column); Anion-exchange chromatography(filled with DEAE-52 cellulose resins); Gel permeation chromatography (Sephadex G-25 column); Size exclusion chromatography(TSK G2000 SWXL column); ESI-MS/MS | *Stichopus japonicus* | Enhancing CD38 inhibitory activity and anti-Aβ aggregation activity | (Lin et al., 2019) |
| sea cucumber peptides | / | / | / | / | / | / | Neutral protease(0.3%, pH = 6.8, 50℃, 2 h) | / | *Stichopus japonicas* | Upregulating the LTP pathway and unsaturating lipid level | (Lu et al., 2022) |
| sea cucumber peptides | / | / | / | / | / | / | / | / | */* | Down-regulating the acetylation level | (Xu et al., 2020) |
| sea cucumber peptides | / | / | / | / | / | / | / | / | */* | Regulating post-transcriptional hippocampal protein acetylation | (Lu et al., 2021b) |
| **Wound Healing** | | | | | | | | | | | |
| VLLY | C_26_H_42_N_4_O_6_ | Val-Leu-Leu-Tyr | 506 Da | (**30**) | / | / | / | / | / | Blocking the binding of MKP to ERK2 and PHLPP to AKT and thus upregulating the ERK/AKT pathway | (Zheng et al., 2022) |
| VTPY | C_23_H_34_N_4_O_7_ | Val-Thr-Pro-Tyr | 479 Da | (**31**) | / | / | / | / | / | Blocking the binding of MKP to ERK2 and PHLPP to AKT and thus upregulating the ERK/AKT pathway | (Zheng et al., 2022) |
| SCCOPs | / | / | <1 kDa | / | rich in Arg, Leu, Tyr, Phe, Lys, and Glu | 89.44% | / | HPLC | *Codonopsis pilosula* | Reducing inflammatory response, improving angiogenesis and collagen deposition, and antioxidation | (Li et al., 2018a) |
| Sh-EGFl-1 | / | / | ~4.9 kDa | / | Cys and Gly | / | / | / | *Stichopus horrens* | Activating the EGFR pathway and inducing cell proliferation via PI3K–AKT–GSK3, Ras–Raf–MEK–ERK–MAPK, PLC gamma, STAT, and Rho signaling pathways | (Pilus et al., 2022) |
| **Anti-hyperuricemic Activity** | | | | | | | | | | | |
| GPAGPR | C_23_H_39_N_9_O_7_ | Gly-Pro-Ala-Gly-Pro-Arg | 553 Da | (**32**) | rich in Gly and Pro | / | Trypsin and alcalase(2:1, 3%, 55℃, 4 h) | MALDI-TOF/TOF | *Apostichopus japonicus* | Modulating the gut microbiota composition and altering the renal miRNA expression in the host | (Fan et al., 2022) |
| GPSGRP | C_23_H_39_N_9_O_8_ | Gly-Pro-Ser-Gly-Arg-Pro | 569 Da | (**33**) | rich in Gly and Pro | / | Trypsin and alcalase(2:1, 3%, 55℃, 4 h) | MALDI-TOF/TOF | *Apostichopus japonicus* | Modulating the gut microbiota composition and altering the renal miRNA expression in the host | (Fan et al., 2022) |
| EH-JAP | / | / | <1 kDa | / | / | / | Trypsin and alcalase(2:1, 3%, 55℃, 4 h) | MALDI-TOF/TOF | *Apostichopus japonicus* | Inhibiting uric acid biosynthesis, promoting uric acid excretion, and inhibiting the activation of TLR4/MyD88/NF-κB signaling pathway | (Wan et al., 2020) |
| EH-LEU | / | / | <1 kDa | / | / | / | Trypsin and alcalase(2:1, 3%, 55℃, 4 h) | MALDI-TOF/TOF | *Acaudina leucoprocta* | Inhibiting uric acid biosynthesis, promoting uric acid excretion, and inhibiting the activation of TLR4/MyD88/NF-κB signaling pathway | (Wan et al., 2020) |
| **Antitumor Effects** | | | | | | | | | | | |
| WPPNYQW | C_50_H_59_N_11_O_11_ | Trp-Pro-Pro-Asn-Tyr-Gln-Trp | 989 Da | (**34**) | / | / | Alcalase(25℃, pH = 7.5, 2 h) | Vacuum filtration(0.22 µm membrane); ultrafiltration；UPLC；LC-MS/MS analysis | *Cucumaria frondosa* | Being stable when EGFR、PI3K、AKT1 and CDK4 complex with the WPPNYQW peptide. | (Wargasetia et al., 2021) |
| YDWRF | C_39_H_47_N_9_O_9_ | Tyr-Asp-Trp-Arg-Phe | 785 Da | (**35**) | / | / | Alcalase(25℃, pH = 7.5, 2 h) | Vacuum filtration(0.22 µm membrane); ultrafiltration; UPLC; LC-MS/MS analysis | *Cucumaria frondosa* | Being stable when YDWRF complex with the  PI3K and AKT1 proteins. | (Wargasetia et al., 2021) |
| sea cucumber intestinal peptide (SCIP) | / | / | < 1 kDa | / | hydrophobicity | / | Alcalase(3%, 50℃, pH = 7, 4 h) | Ultrafiltration(< 1 kDa); RP-HPLC | *Atlantic Sea cucumber* | Inhibiting PI3K/AKT signaling Pathway | (Wei et al., 2021) |
| Sea cucumber peptides | / | / | / | / | / | / | 1000 U complex protease (papain : trypsin = 2 : 1, 40℃, 5 h) | Alcohol precipitation | *Russian polar ginseng* | Regulating miR-378a-5p targeted TUSC2 | (Mao et al., 2021) |
| **Anti-fatigue Properties** | | | | | | | | | | | |
| Sea cucumber peptides | / | / | <2 kDa | / | rich in Gly, Glu, and Pro | / | Neutral protease(1 : 100(w/w), 50℃, pH = 7, 4 h) | Ultrafiltration(<5 kDa); high performance size exclusion chromatography(SRT SEC-100 column) | *Stichopus japonicas* | Normalizing energy metabolism as well as alleviating oxidative damage and inflammatory responses. | (Ye et al., 2017) |
| Apostichopus Japonicus Oligopeptides | / | / | <1 kDa | / | rich in Gly, Glu, Asp, Ala, HYP, Pro and Leu. | > 98 % | Protamex | High performance gel filtration chromatography | *Apostichopus japonicus* | Reducing the content of serum urea nitrogen and increasing the reserve capacity of glycogen *in vivo* | (Wang et al., 2019) |
| Sea cucumber peptides | / | / | <5 kDa | / | rich in Asp, Glu, Gly, Arg, and Pro | / | Neutral protease (1 : 100(w/w), 50℃, pH = 7, 4 h) | Ultrafiltration(<5 kDa); HPLC(TSKGel 2000 SWXL) | *Stichopus japonicus* | Enhancing mitochondrial function | (Yu et al., 2020) |
| **Hypoglycemic Activity** | | | | | | | | | | | |
| GDFs | / | / | <3 kDa | / | / | / | Pepsin(2 kU.mL^-1^, 37℃, pH = 3, 2 h) | Molecular weight cut-off microsep(<3 kDa); LC-MS/MS(C18 capillary trap column); [Molecular Size Distribution](https://www.sciencedirect.com/topics/engineering/molecular-size-distribution)(16.5% Tricine-SDS-PAGE) | *Stichopus japonicus* | Inhibiting the target enzyme DPP-IV | (Gong et al., 2020) |
| IDFs | / | / | <3 kDa | / | / | / | Pepsin(2 kU.mL^-1^, 37℃, pH = 3, 2 h)→trypsin and chymotrypsin(100 U.mL^-1^ and 25 U.mL^-1^, pH = 7.0) | Molecular weight cut-off microsep(<3 kDa) LC-MS/MS(C18 capillary trap column); [Molecular Size Distribution](https://www.sciencedirect.com/topics/engineering/molecular-size-distribution)(16.5%Tricine-SDS-PAGE) | *Stichopus japonicus* | Inhibiting the target enzyme DPP-IV | (Gong et al., 2020) |
| Apostichopus japonicus hydrolysate | / | / | <3kDa | / | / | / | Trypsin(2.02%, 51.18°C, 127.81 min) | MALDI-TOF / TOF | *Apostichopus japonicus* | Inhibiting the MAPK and p38MAPK signaling pathways | (Dong et al., 2018) |
| peptide fractions （PF） | / | / | / | / | rich in Asp, Arg, Glu, Lys, Cys, and Ser | / | Protamex(1.8%(w/v), 1.0 × 10^5^ U.g^-1^, 55℃, 136 min) | / | *Apostichopus japonicus；Acaudina molpadioides* | / | (Li et al., 2017) |
| **Promotion of Bone Growth** | | | | | | | | | | | |
| sea cucumber intestines (SCIP) | / | / | 200-1 000Da | / | Glu, Asp, Gly, and Gln | 68.8% | Alcalase (2%(w/w), pH = 9.0, 55℃, 5 h) | Size-exclusion chromatography | */* | Accelerating cell cycle progress by regulating glutamine metabolism | (Yue et al., 2022a) |
| sea  cucumber intestine (SCIP) | / | / | <3 kDa | / | / | / | Alcalase (2%(w/w), pH = 9.0, 55℃, 5 h) | Ultrafiltration(<3 kDa); Purification(Sephadex G-15 column); LC−MS/MS(ACQUITY UPLC Peptide CSH C18 column) | */* | Redifferentiating to osteoblast via integrin-mediated Wnt/β-catenin signaling | (Yue et al., 2022b) |
| **Promotion of Collagen Synthesis and Secretion** | | | | | | | | | | | |
| SP12 | / | / | ~3.87 kDa | / | / | 97.4% | Protamex(Alcalase : Papain：trypsin = 3 : 3 : 4, 1%, pH = 7.0, 12 h) | Ultrafiltration(< 6 kDa); ion exchange chromatography(DEAE Anion exchange chromatography column); gel filtration chromatography(S200 gel column) | *Stichopus japonicus* | Promoting the expression of type I collagen and TIMP-1, and inhibiting the expression of MMP-1 | (Song et al., 2017) |
| Sea cucumber peptides | / | / | <5 kDa | / | / | / | Animal proteinase and neutral protease(w /w = 1 : 1(g /g), v /w = 3 : 1(mL /g), 4%, 50℃, 4 h) | Ultrafiltration(<5 kDa) | *Acaudina leucoproata* | Accelerating the cell proliferation and collagen production | (Jiang et al., 2014) |
| **Anti-inflammation** | | | | | | | | | | | |
| Sea cucumber peptides | / | / | 180-1 000 Da | / | Rich in Gly, Glu, and Asp | 72.12% | Flavor protease(5%, pH = 7.0, 50℃, 8 h) | Gel filtration chromatography(Sephadex G-25 column); size-exclusion chromatography(TSK gel chromatographic column) | */* | Up-regulating the expression of HO-1 and inhibiting inflammation | (Song et al., 2016) |
| AJH-1 | / | / | <10 kDa | / | Val, Ala-Pro-Arg, Gly-Lys,  Asp propyl ester, Glu methyl ester, His butyl ester, Ile-Ala-Ala-Lys, Tyr-Lys, and  Asn-Pro-Gly-Lys. | / | Trypsin(1 kU.g^-1^, 35℃, 1 h) | Ultrafiltration(<10 kDa); LC-MS/MS(Agilent Eclipse XDB-C18 column) | *Apostichopus japonicus* | Leukocyting migration to the site of injury is blocked. | (Zhang et al., 2021) |
| **Immunological Regulation** | | | | | | | | | | | |
| SOP | / | / | < 1 kDa | / |  | 89.44% | / | HPLC(Phenomenex C18 column); MALDI-TOF-MS | *Codonopsis pilosula* | Stimulating Th cells, cytokine secretion and antibody production | (He et al., 2016) |

“ * “represents the structure of the peptides that have been explicitly mentioned in the reference.
